# Toxin Release of Cyanobacterium *Microcystis aeruginosa* after Exposure to Typical Tetracycline Antibiotic Contaminants

**DOI:** 10.3390/toxins9020053

**Published:** 2017-02-21

**Authors:** Jing Ye, Yuping Du, Lumei Wang, Jingru Qian, Jiejing Chen, Qingwen Wu, Xiaojun Hu

**Affiliations:** 1 School of Chemical and Environmental Engineering, Shanghai Institute of Technology, Shanghai 201418, China; 18531139640@163.com (Y.D.); good2009314@163.com (J.Q.); sonia0910@163.com (J.C.); wuqingwen1990@126.com (Q.W.); 2 Key Laboratory of Urban Agriculture (South) of Ministry of Agriculture, School of Agriculture and Biology, Shanghai Jiao Tong University, Shanghai 200240, China; zjuwlm@163.com

**Keywords:** toxin release, blue-green algae, tetracycline antibiotics, environmental toxicology

## Abstract

The global usage of veterinary antibiotics is significant. Antibiotics can be released into aquatic environments and elicit toxic effects on non-target organisms. In this study, the growth characteristics and toxin release of the cyanobacterium *Microcystis aeruginosa* (*M. aeruginosa*) were examined to investigate the physiological effects of tetracycline antibiotics on aquatic life. Results showed that the degree of toxicities of the following target antibiotics was TC (tetracycline hydrochloride) > CTC (chlortetracycline hydrochloride) > OTC (oxytetracycline hydrochloride) in terms of growth parameters, EC_10_ (0.63, 1.86, and 3.02 mg/L, respectively), and EC_20_ (1.58, 4.09, and 4.86 mg/L, respectively) values. These antibiotics inhibited the production of microcystin-LR (MC-LR) to varying degrees. CTC interfered *M. aeruginosa* cells and decreased their ability to release MC-LR, but this antibiotic stimulated the ability of these cells to synthesize MC-LR at 2 and 5 mg/L. OTC elicited a relatively weaker toxicity than CTC did and reduced MC-LR release. TC was the most toxic among the three antibiotics, and this antibiotic simultaneously reduced intracellular and extracellular MC-LR equivalents. Our results helped elucidate the effects of tetracycline antibiotics on *M. aeruginosa*, which is essential for environmental evaluation and protection. Our results are also helpful for guiding the application of veterinary antibiotics in agricultural settings.

## 1. Introduction

Veterinary antibiotics are biologically active molecules that are widely administered as therapeutics and growth promoters in livestock production and feed additives in fish farms; these molecules are also used to prevent crop damage induced by bacteria [1]. According to the European Federation of Animal Health, more than 4,700 tonnes of antibiotics were administered to farm animals in 1999 in the European Union (EU) [2]. The annual consumption of antibiotics by livestock in the USA is approximately 11,000 tonnes [3]. In China, 15,770 tonnes of antibiotics were used as non-prescription therapeutics in 2004 [4]. The annual global usage of antibiotics has been estimated between 100,000 and 200,000 tonnes globally [5], and this amount has increased in many developed and developing nations.

Most antibiotics are water soluble, and various fractions (5%–90%) are discharged into aquatic environments as parent compounds [6]. It has been estimated that wastewater treatment plants remove 80% of total antibiotics from wastewater, but only 30% of tetracycline antibiotics [7]. Antibiotics are regarded as emerging “pseudopersistent” environmental pollutants because of their continuous input and persistence in aquatic ecosystems. Cases of surface water contamination by antibiotics have been reported since 1982 [8]. Antibiotics are also widely distributed in aquatic environments, and their concentrations in contaminated freshwaters range from ng/L to μg/L. In certain cases, their concentrations can reach up to 50 mg/L in locally contaminated sources in production facilities [9].

Antibiotics are specifically designed to affect biological systems, easily penetrate biomembranes, avoid biodegradation, and become as effective as possible [10]. These lipophilic properties suggest that antibiotics can accumulate in food chains and freshwater. For example, oxytetracycline (OTC) has a half-life of more than 300 days in marine sediments [10]. However, antibiotic pollutants target aquatic organisms because they are exposed to these compounds throughout their lifetime [11]. Because their cell structure is similar to bacteria [12], cyanobacteria are generally more sensitive to antibiotic contaminants compared with other algal species, and it can be served as an indicator species of water toxicity [13]. Antibacterial agents are highly toxic to the cyanobacterium *Microcystis aeruginosa* (*M. aeruginosa*), and this prokaryote must be included as an aquatic toxicity test species. Considering the sensitivity of cyanobacteria, the European Medicines Evaluation Agency recommended the use of these organisms for the evaluation of the effects of antimicrobials [14]. Antibiotic contaminants can affect the growth and photosynthetic efficiency of *M. aeruginosa* [15,16,17,18,19], modify photosynthesis-related gene transcription, and disrupt the balance between oxidant substances and antioxidant enzymes in *M. aeruginosa* [5]. Antibiotics also influence the production and release of microcystins [20]. In addition, antibiotics exert physiological effects on cyanobacteria, which are primary producers, and potentially disrupt ecosystem processes. Therefore, the effects of antibiotics on cyanobacteria should be monitored and evaluated. Unfortunately, relevant ecotoxicity data for many antibiotics are unavailable, and the knowledge of the potential effects of antibiotics on the environment is limited.

In veterinary medicine, tetracyclines exhibit a broad spectrum of antibacterial activity for the treatment and control of various bacterial infections [21]. Tetracyclines pass through sensitive bacterial cells through active transport and bind the 30S subunit of ribosomes. Consequently, the binding of aminoacyl transfer RNA to DNA is prevented and protein synthesis and bacterial growth are inhibited. Tetracyclines are also the most highly consumed antibiotics in the EU [22]. Our study aimed to evaluate the toxic effects of tetracycline antibiotics on aquatic organisms to obtain information on the risks involved in the release of these drugs into the environment. *M. aeruginosa* was exposed to three widely used antibiotics, namely, oxytetracycline hydrochloride (OTC), chlortetracycline hydrochloride (CTC), and tetracycline hydrochloride (TC), in controlled laboratory experiments. The growth characteristics of *M. aeruginosa* and its production and release of microcystins (MC-LR equivalents) were examined.

## 2. Results

### 2.1. Exposure Concentrations of the Chemicals

For the nominal concentrations of 1, 2, 5, 10, and 20 mg/L, the exposure concentrations of CTC were 0.97 ± 0.39, 1.59 ± 0.32, 4.65 ± 0.86, 11.04 ± 1.72, and 23.31 ± 2.88 mg/L, respectively. For the nominal concentrations of 1, 2, 5, and 10 mg/L, the exposure concentrations of OTC were 0.87 ± 0.11, 1.75 ± 0.15, 4.59 ± 0.09 and 9.36 ± 0.08 mg/L, respectively. For the nominal concentrations of 1, 2, 5, and 10 mg/L, the exposure concentrations of TC were 0.97 ± 0.10, 1.44 ± 0.22, 3.62 ± 0.12, and 8.33 ± 0.15 mg/L, respectively.

### 2.2. Growth Curves of M. aeruginosa

**Figure 1:**
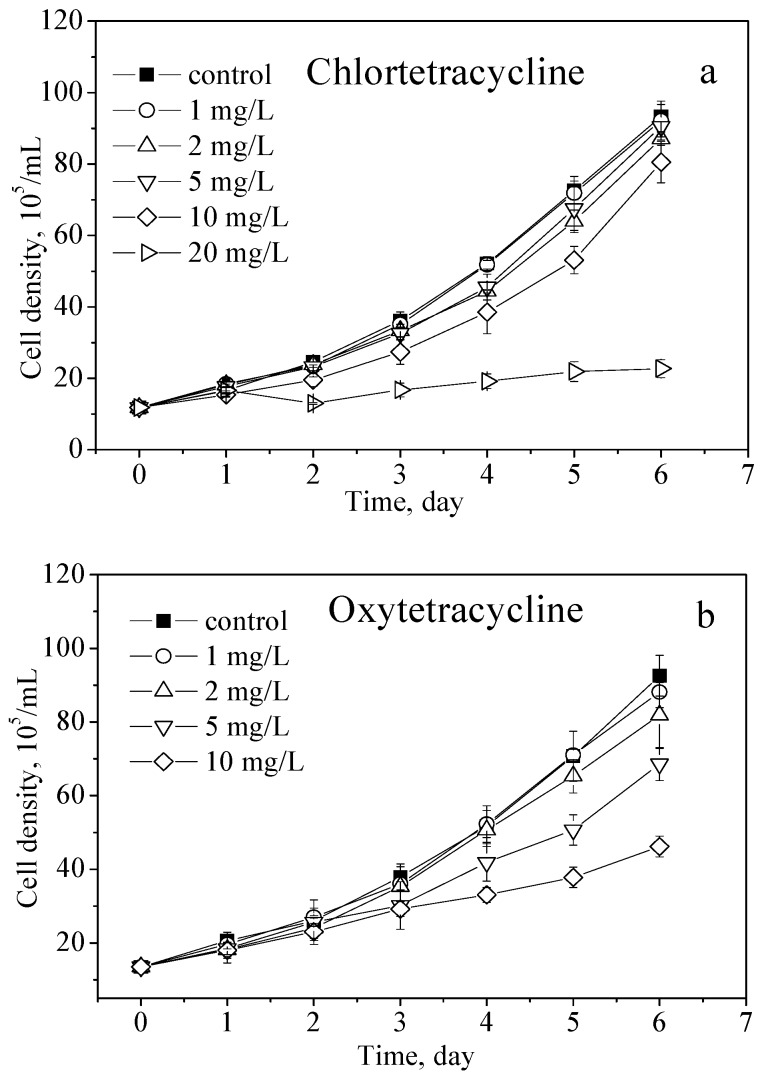

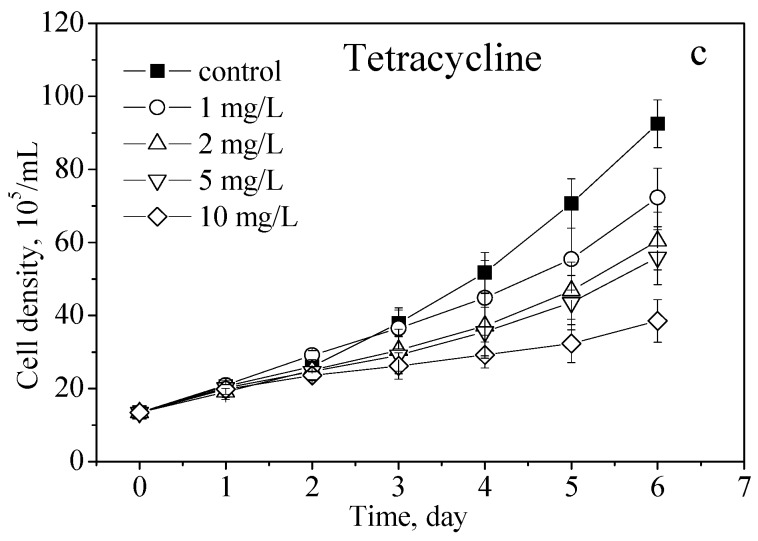
Growth curves of *M. aeruginosa*. The results are presented as mean ± SD of triplicates. **a–c** are for chlortetracycline hydrochloride, oxytetracycline hydrochloride, and tetracycline hydrochloride, respectively.

The growth curves of *M. aeruginosa* exposed to CTC, OTC, and TC are shown in Figure 1a–c. At 1 mg/L, the cell density was not significantly affected by CTC during the experiment (Figure 1a). After day 4 at 2 mg/L, after day 3 at 5 mg/L, and after day 2 at 10 and 20 mg/L, the growth of *M. aeruginosa* was significantly inhibited. The inhibition percentages on day 6 were 6.74%, 3.07%, 13.73%, and 75.68% for 2, 5, 10, and 20 mg/L, respectively. In general, the inhibitory effects were related to compound concentration. At 20 mg/L, 75.8% of the growth of *M. aeruginosa* was significantly inhibited by CTC.

Similar to the growth curve of *M. aeruginosa* exposed to CTC, the growth curve of *M. aeruginosa* exposed to 1 mg/L OTC was not significantly affected (Figure 1b). At 2 mg/L, a significant inhibitory effect was observed after day 5. The OTC concentrations of 5 and 10 mg/L significantly inhibited the growth of *M. aeruginosa* after day 3. The inhibition percentages on day 6 were 11.57%, 25.85% and 50.10% for 2, 5, and 10 mg/L, respectively. Similar to those of CTC, the inhibitory effects of OTC were related to compound concentration.

By contrast, TC significantly inhibited the growth of *M. aeruginosa* at 1 mg/L after day 4 (Figure 1c). The TC concentrations of 2, 5, and 10 mg/L significantly inhibited the growth of *M. aeruginosa* after day 3. The inhibition percentages on day 6 were 21.81%, 34.72%, 39.53%, and 58.37% for 1, 2, 5, and 10 mg/L, respectively. These inhibitory effects were also related to compound concentration.

At the end of the experiment (day 6), all of the TC concentrations inhibited more than 20% of cell growth. By comparison, 5 mg/L OTC and 20 mg/L CTC inhibited more than 20% of cell growth. In addition, 10 mg/L OTC and TC and 20 mg/L CTC inhibited more than 50% of cell growth.

The 96 h EC_10_ and EC_20_ of the three antibiotics toward *M. aeruginosa* were calculated, and the results are presented in Table 1. The EC_10_ and EC_20_ values of TC, CTC, and OTC demonstrated that the toxicity order of the three target antibiotics was TC > CTC > OTC. Logistic regression was applied to describe the growth mode of *M. aeruginosa* exposed to different concentrations of the three tetracyclines (Table 2). The results indicated that the parameter k is related to the exposure concentration. With an increase in the concentration of the antibiotics, the parameter k decreased. For CTC, the parameter k significantly decreased from 0.62 to 0.48 (except 20 mg/L). This finding suggested that the growth of *M. aeruginosa* was inhibited by CTC. The effects of OTC and TC were similar to those of CTC.

### 2.3. ELISA Detection of MC-LR in the CTC-Treated Samples

**Figure 2:**
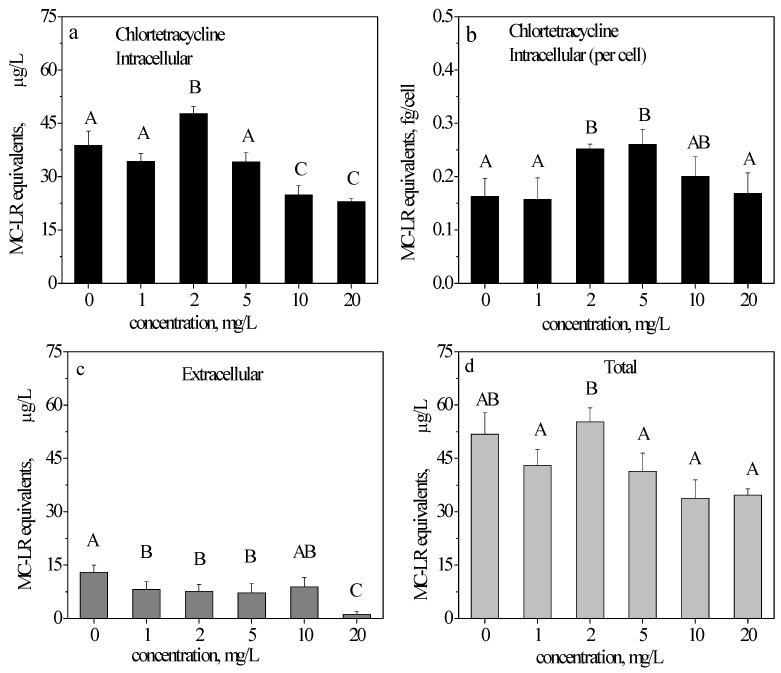
Intracellular, extracellular and total microcystin-LR (MC-LR) equivalents in *M. aeruginosa* cells exposed to chlortetracycline hydrochloride (CTC). Intracellular (**a**,**b**), extracellular (**c**), and total (**d**) MC-LR equivalents in *M. aeruginosa* cells exposed to 0, 1, 2, 5, 10, and 20 mg/L CTC after 48 h. The results are presented as the mean ± SD of triplicates. The different capitalized letters above adjacent bars indicate a significant difference (*p* < 0.05) between the individual concentrations, while the same letter indicates no significant difference (LSD).

After a 48-h exposure to CTC compared with the control, the intracellular production of MC-LR equivalents significantly increased by 22.68% at 2 mg/L and decreased by 35.82% and 40.72% at 10 and 20 mg/L, respectively. At 5 mg/L, the MC-LR production was not significantly different compared with the control (Figure 2a). For each cell’s MC-LR production, compared with the control, the result at 2 mg/L was similar to the intracellular MC-LR production with an increased percentage of 54.67% (Figure 2b), but was different at 10 and 20 mg/L, which indicated no significant differences. In addition, at 5 mg/L, the MC-LR production in each cell significantly increased by 60.01%.

The extracellular MC-LR equivalents (Figure 2c) were significantly lower than the intracellular MC-LR equivalents. Therefore, it can be inferred that at 48 h, most cells were intact, and most of the extracelluar toxin was released by living cells. At 1, 2, 5, and 20 mg/L, the release of MC-LR decreased significantly compared with the control.

The total MC-LR (intracellular + extracellular) results are shown in Figure 2d. The total MC-LR equivalents decreased by 16.99%, 20.27%, 34.75, and 33.01% at 1, 5, 10, and 20 mg/L, respectively.

### 2.4. ELISA Detection of MC-LR in the OTC-Treated Samples

**Figure 3:**
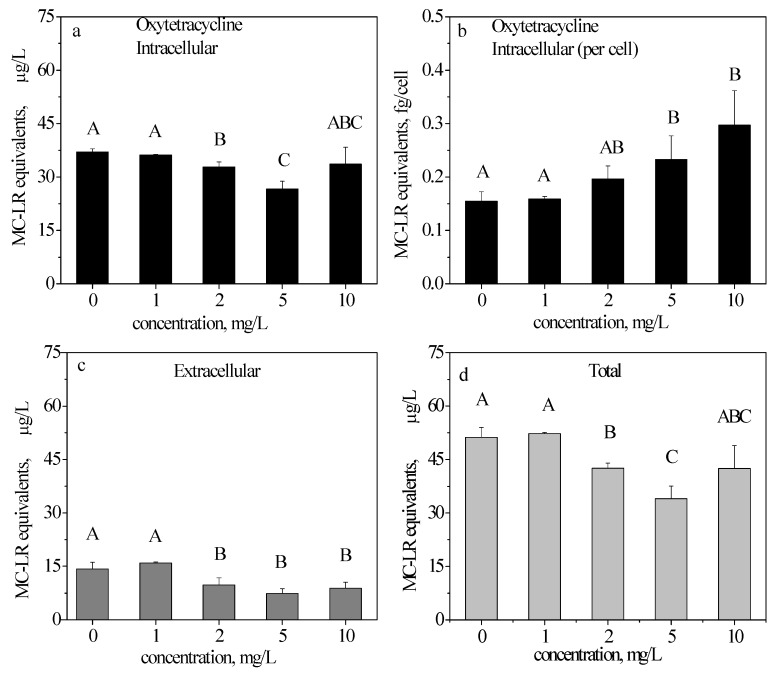
Intracellular, extracellular and total microcystin-LR (MC-LR) equivalents in *M. aeruginosa* cells exposed to oxytetracycline hydrochloride (OTC). Intracellular (**a**,**b**), extracellular (**c**), and total (**d**) MC-LR equivalents in *M. aeruginosa* cells exposed to 0, 1, 2, 5, and 10 mg/L OTC after 48 h. The results are presented as the mean ± SD of triplicates. The different capitalized letters above adjacent bars indicate a significant difference (*p* < 0.05) between the individual concentrations, while the same letter indicates no significant difference (LSD).

After a 48 h exposure to OTC compared with the control, the intracellular production of MC-LR decreased significantly by 11.35% and 28.11% at 2 mg/L and 5 mg/L, respectively. At 10 mg/L, the MC-LR production was not significantly different compared with the control (Figure 3a). However, for each cell’s MC-LR production, the results were different, with increasing percentages of 26.73%, 50.48%, and 91.80% at 2, 5, and 10 mg/L compared with the control, respectively (Figure 3b).

Similar to CTC, the extracellular MC-LR equivalents (Figure 3c) were significantly lower than the intracellular MC-LR equivalents after exposure to OTC. At 2, 5, and 10 mg/L, the release of MC-LR decreased significantly by 31.56%, 48.20% and 38.13% compared with the control, respectively.

The total MC-LR (intracellular + extracellular) results are shown in Figure 3d. The total MC-LR equivalents decreased by 16.96%, 33.60% and 17.11% at 2, 5, and 10 mg/L, respectively.

### 2.5. ELISA Detection of MC-LR in the TC-Treated Samples

**Figure 4:**
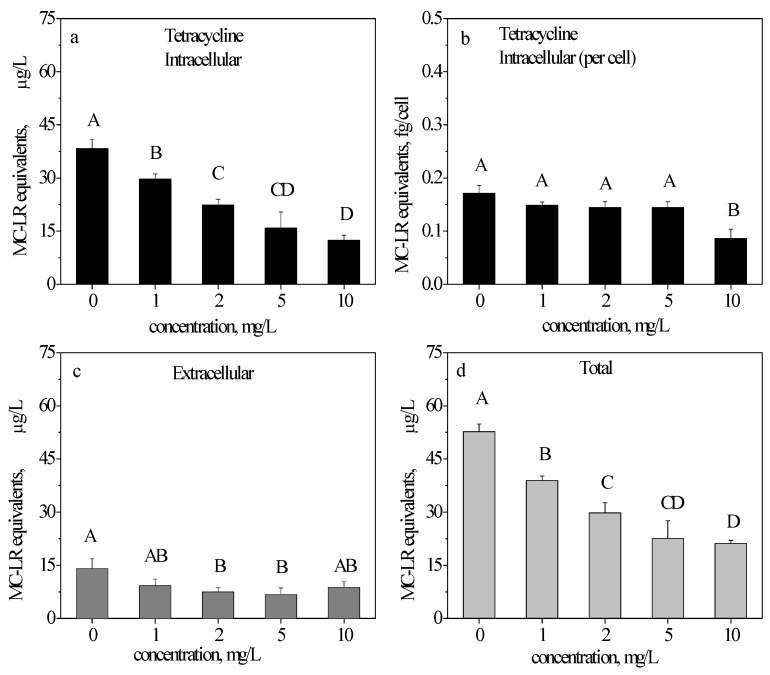
Intracellular, extracellular, and total microcystin-LR (MC-LR) equivalents in *M. aeruginosa* cells exposed to tetracycline hydrochloride (TC). Intracellular (**a**,**b**), extracellular (**c**), and total (**d**) MC-LR equivalents in *M. aeruginosa* cells exposed to 0, 1, 2, 5, and 10 mg/L TC after 48 h. The results are presented as the mean ± SD of triplicates. The different capitalized letters above adjacent bars indicate a significant difference (*p* < 0.05) between the individual concentrations, while the same letter indicates no significant difference (LSD).

After a 48 h exposure to TC compared with the control, the intracellular production of MC-LR decreased significantly by 22.65%, 41.88%, 58.68% and 67.52% at 1, 2, 5, and 10 mg/L, respectively (Figure 4a). This decrease is related to the concentration. For the MC-LR production in each cell, the results were different, with a decreasing percentage of 49.47% only at 10 mg/L (Figure 4b) compared with the control.

Similar to CTC and OTC, the extracellular MC-LR equivalents (Figure 4c) were significantly lower than the intracellular MC-LR equivalents after exposure to TC. At 1, 2, 5, and 10 mg/L, the release of MC-LR decreased by 34.41%, 46.99%, 52.41% and 38.13% compared with the control, respectively.

The total MC-LR (intracellular + extracellular) results are shown in Figure 4d. The total MC-LR equivalents decreased by 26.19%, 43.54%, 57.22%, and 59.76% at 1, 2, 5, and 10 mg/L, respectively.

As indicated in Figure 2a, Figure 3a and Figure 4a, with an increasing concentration, CTC initially stimulated, and then inhibited the intracellular production of MC-LR; OTC inhibited the production of MC-LR only at 5 mg/L, while TC inhibited the production of MC-LR. Figure 2b, Figure 3b and Figure 4b illustrate the ability of MC-LR production. CTC stimulated the production of MC-LR in each cell at 2 and 5 mg/L (in accordance with Figure 2a); OTC also stimulated the production of MC-LR in each cell above 2 mg/L (different from Figure 3a); as opposed to CTC and OTC, TC only inhibited the production of MC-LR in each cell at 10 mg/L.

The total extracellular content of the three antibiotics (Figure 2c, Figure 3c and Figure 4c) were all decreased. The total MC-LR (Figure 2d, Figure 3d and Figure 4d) behaved similarly to intracellular MC-LR (Figure 2a, Figure 3a and Figure 4a). Our results suggested that TC was the most toxic among the three antibiotics. TC inhibited the production of MC-LR (Figure 4a) and the ability to synthesize MC-LR (Figure 4b).

## 3. Discussion

### 3.1. Toxicity of the Antibiotics to M. aeruginosa

Veterinary antibiotics have shown to release into the aquatic environment and exert toxic effects on non-target organisms. Generally, antibiotics are considered moderately toxic to aquatic invertebrates or fish [23,24], and a broad range of antibiotics exhibit a different toxicity to non-target organisms. Lanzky and Halling-Sørensen [10] have shown that *Chlorella* sp. is highly sensitive (EC_10_ value of 2.03 mg/L) to the antibiotic metronidazole. Acute toxicity (LC_50_) of furazolidone was found at 40 mg/kg for the mosquito larvae *Culex pipiens* [25]. Amoxicillin exerts different toxicities on different species of microalgae and aquatic plants [20].

Currently, according to the results obtained by Lanzky and Halling-Sørensen [10], we found that *M. aeruginosa* is also sensitive to tetracyclines (with EC_10_ values of 0.63, 1.86, and 3.02 mg/L for TC, CTC, and OTC, respectively). As shown in Figure 1, all of the three tested antibiotics inhibited the growth of *M. aeruginosa* under the experimental conditions, and the inhibitory effects are related to the concentration of the antibiotics. With an increasing concentration, the inhibition occurred earlier. Regarding the inhibition occurrence time, the toxicity order was TC > CTC > OTC. EC_10_ and EC_20_ values from Table 1 demonstrated the same toxicity order. From the results of the logistic growth model (Table 2), the parameter k, which represents the growth rate, decreased with increasing concentration, and at 1, 2 and 10 mg/L, the values were in the order of k_TC_ ≤ k_CTC_ < k_OTC_, which demonstrated the same toxicity order. Slight changes in the chemical structures of the antibiotics may markedly affect the antibacterial properties and may be the cause of the observed differences [6]. Although the three antibiotics belong to the same class, they elicit different degrees of toxic effects on *M. aeruginosa*.

Guo and Chen [18] reported that the growth of *M. aeruginosa* was inhibited by CTC at 1 mg/L. Van der Grinten et al. [15] indicated that EC_50_ value of OTC in *M. aeruginosa* is 5.4 mg/L and Kolar et al. [17] reported that EC_50_ and EC_10_ of OTC in *Anabaena flos-aque* were 2.7 and 1.5 mg/L, respectively. Ando et al. [26] and Holten Lützhøft et al. [27] revealed that EC_50_ value of OTC in *M. aeruginosa* is 0.207–0.23 mg/L, which is lower than that obtained in our present study (EC_20_ of OTC in our present study was 4.86 mg/L). This result may be attributed to the lower initial cell density of 2 × 10^4^ cells/mL used, which possibly led to the greater sensitivity. González-Pleiter et al. [19] demonstrated that TC is toxic to the cyanobacteria *Anabaena* sp. CPB4337 with an EC_10_ value of 2.5 ± 0.7 mg/L. Halling-Sørensen et al. [13] showed that EC_50_ value of TC in *M. aeruginosa* is 0.09 mg/L. Dias et. al. [16] indicated that the minimum inhibitory concentrations of TC on *M. aeruginosa* are higher than 1.6 mg/L. The discrepancies between our results and those in previous studies may be due to the different endpoints used to measure growth, which might influence the outcome [16]. In most published studies (except Dias et al.), the growth inhibitions were monitored by measuring chlorophyll fluorescence or chlorophyll-a content; however, in this study the results were obtained from cell density. Therefore, further research should be performed.

Cyanobacteria are photoautotrophic organisms in aquatic ecosystems. The observed toxicity exerted by certain antibiotics may affect the food chain.

### 3.2. Influence of Antibiotics on MC-LR Production and Release in M. aeruginosa

MC-LR is one of the predominant MC variants in water blooms. As indicated in Figure 2a, with an increasing concentration, CTC initially stimulated (at 2 mg/L) and then inhibited (at 10 and 20 mg/L) the intracellular production of MC-LR. The synthesis of toxins is regarded as a protective mechanism against predators, environmental stresses, and competition from other species [28]. The increased production of MC-LR may be in response to the stress caused by CTC under 2 mg/L. Figure 2b better illustrates the ability to synthesize MC-LR in *M. aeruginosa*, which indicated that the ability to synthesize MC-LR was stimulated under 2 and 5 mg/L. The total extracellular MC-LR equivalents (Figure 2c) significantly decreased under the high concentration of 20 mg/L. From the growth curves in Figure 1, it can be inferred that under a high CTC concentration exposure, the cell was destroyed, which resulted in a decrease in the MC-LR release, but the ability to synthesize MC-LR was not affected.

OTC did not inhibit considerably the growth of *M. aeruginosa* after 48 h of exposure to the tested concentrations. However, the production of MC-LR under 2 and 5 mg/L (Figure 3a) was inhibited, and the toxin cell quota increased above 2 mg/L (Figure 3b). OTC interferes more with toxin production than that of *M. aeruginosa* growth. Under laboratory conditions, OTC elicited a relatively weaker toxicity than CTC did. The extracellular MC-LR equivalents of MC-LR decreased at 2, 5, and 10 mg/L. This result does not indicate that OTC is less noxious to the aquatic environment because the increased production of MC-LR in the cells may ultimately be released into the surrounding water when the blooms decay.

TC inhibited the production of MC-LR (Figure 4a) and the ability to synthesize MC-LR (Figure 4b). On the basis of the growth curves and parameters, we conclude that TC is the most toxic antibiotic to *M. aeruginosa*.

The MC-LR production was antibiotic-dependent. CTC induced an increase in MC-LR production at 2 and 5 mg/L (Figure 2b) while OTC made the increase at above 2 mg/L (Figure 3b). Conversely, TC induced a decrease in MC-LR cell quota at 10 mg/L (Figure 4b). The toxin remained mainly in the intracellular fraction at 48 h of exposure, because at this time point the antibiotics uninduced significant effects on cell viability. In addition, from Figure 2a,b, Figure 3a,b and Figure 4a,b, the variation tendencies of Figure 2a,b (CTC) and Figure 4a,b (TC) were almost the same. Therefore, CTC and TC simultaneously affected on the production of MC-LR and cell growth. However, variation tendencies of Figure 3a,b (OTC) were different, thereby indicating that OTC interferes more with toxin production than that of cell growth.

*M. aeruginosa* is a ubiquitous cyanobacterium often linked to toxic blooms worldwide [29]. High concentrations of *Microcystis* do not necessarily correlate with high levels of MCs and other toxins in the water column. Many cyanobacteria retain cyanotoxins within their cell structure and only release these toxins into the surrounding water upon cell lysis [30]. Certain environmental factors, such as pH, temperature, and the amount of phosphorus and nitrogen, have been found to affect the production of MCs [31]. Other factors, such as environmental pollutants, have rarely been studied. Previously, we demonstrated that the herbicide diclofop acid can affect the production and release of MC-LR in *M. aeruginosa* [32]. Liu et al. [20] reported that spiramycin and amoxicillin can affect the production and release of MCs in *M. aeruginosa*. The present study found that CTC, OTC and TC interfered the production of MC-LR to different degrees, with different manners, and most MC-LR equivalents mainly in the intracellular fraction at 48 h of exposure.

Tetracycline antibiotics prevent the binding of aminoacyl transfer RNA to DNA and inhibit protein synthesis and bacterial growth by binding the 30S subunit of the ribosomes. The synthesis of MC-LR is controlled by peptide synthetases [33]. Tetracycline antibiotics may inhibit the production of MC-LR in *M. aeruginosa* by blocking the synthesis of the peptide synthetases in the ribosome, thereby leading to the decreased MC-LR equivalents. TC is the most toxic antibiotic; it significantly inhibited the production and release of MC-LR. The chemical structure of TC is simpler than those of OTC and CTC; therefore, TC may enter the cell membrane more efficiently than CTC and OTC. However, the mechanism demands further research. The inhibition of the cell growth at a 48 h exposure (Figure 1a, 10 and 20 mg/L; Figure 1b, 2, 5, and 10 mg/L; Figure 1c, 1, 2, 5, and 10 mg/L) also contributed to the decreased production of MC-LR.

## 4. Conclusions

In conclusion, tetracycline antibiotics were demonstrated to exert toxic effects on the non-target organism *M. aeruginosa*. The following results were obtained. (1) *M. aeruginosa* is sensitive to the tetracycline antibiotics CTC, OTC, and TC, and the degree of toxicity was TC > CTC > OTC; (2) The target antibiotics interfered the production of MC-LR to varying degrees. Among these antibiotics, TC was the most toxic, and it inhibited the production of MC-LR and the ability of *M. aeruginosa* to synthesize MC-LR. As a result, its extracellular MC-LR equivalents decreased. Therefore, TC simultaneously reduced the intracellular and extracellular MC-LR equivalents. The study of the ecological effects of pharmaceuticals on the environment is in an intermediate period [34]. Note that there is a conspicuous lack of data on the presence of antibiotics in the environment [23,35,36,37,38], leaving many possibilities for future explorations in this field. Our study contributes to the understanding of the inference of antibiotics on toxic cyanobacteria.

## 5. Materials and Methods 

### 5.1. Chemicals and Cell Culture

CTC (chlortetracycline hydrochloride, 93% purity, water solubility = 8.6 g/L), OTC (oxytetracycline hydrochloride, 96% purity, water solubility > 100 g/L), and TC (tetracycline hydrochloride, 98% purity, water solubility = 50 g/L) were purchased from Dr. Ehrenstorfer GmbH (Augsburg, Germany). Standard MC-LR, with a purity ≥95% was purchased from Express Technology Co., Ltd. (Beijing, China).

The cyanobacterium *M. aeruginosa* (axenic strain, FACHB 905) was obtained from the Freshwater Algae Culture Collection of the Institute of Hydrobiology in China. The unialgal inoculant (axenic strain) was cultured in sterile BG11 medium under an irradiance of 40 µmol/m^2^·s with a wavelength ranging from 400 to 750 nm and a photoperiod of 12 h light/12 h dark at 28 ± 1 °C.

### 5.2. Growth Characteristic Tests

In our present work, the test concentrations used were at the mg/L level (much higher than realistic concentrations) to obtain toxicity data, which is necessary for the risk assessment of tetracycline antibiotics. The growth characteristic tests were performed using 0, 1, 2, 5, 10, and 20 mg/L CTC and 0, 1, 2, 5, and 10 mg/L OTC and TC. The 3 replicates of each concentration were prepared in 100 mL Erlenmeyer flasks, thereby containing 5 mL of the algal inoculant and 45 mL of culture medium. The linear equation between the cell number and the optical density of the algal culture at 685 nm was established using a UV/vis spectrometer (LabTech, Beijing, China). The initial algal density in each flask was (1.1–1.4) × 10^6^ cells/mL. The algal cultures were shaken three times per day. The algal densities were measured every 24 h for 6 days to obtain the growth curve under different conditions. The following logistic regression (Equation (1)) was applied to describe the growth mode of *M. aeruginosa*:

(1) $$N_{t}=\frac{N_{F}}{1+e^{-k(t-tc)}}$$

where *N_t_* represents the cell number of *M. aeruginosa*, *N*_F_ represents the final cell number of *M. aeruginosa* during the experimental period, and tc represents the time when *N_t_* = $\frac{N_{F}}{2}$.

The cell growth-inhibition tests were carried out according to the OECD guidelines for the testing of chemicals (201) [39]. The EC_10_ and EC_20_ values were calculated after 96 h of exposure. The inhibition of growth is expressed as the logarithmic increase in biomass (average specific growth rate) during 96 h of exposure. From the average specific growth rates recorded in a series of test solutions, the concentration bringing about a specified 10% or 20% inhibition of growth rate is determined and expressed as the EC_10_ or EC_20_.

### 5.3. Analysis of the Exposure Concentrations

After the chemicals were added into the culture medium, triplicate culture samples were filtered through a 0.45 μm filter and analyzed by HPLC. The analyses were performed on a LabTech LC 600 plus HPLC system (LabTech, Beijing, China) with a P600 high-pressure constant flow pump, a UV600 UV/vis detector, and a Promosil C18 column (4.6 mm × 250 mm, LabTech, Beijing, China). For the detection of CTC, the operation conditions were a flow rate of 1.0 mL/min, a mobile phase of 0.01 M oxalic acid/acetonitrile/methanol (67:20:13, *v*/*v*/*v*), a detection wavelength of 360 nm, and an injection volume of 10 μL at room temperature. For the detection of OTC, the operation conditions were a flow rate of 1.0 mL/min, a mobile phase of 0.05 M potassium dihydrogen phosphate/acetonitrile (18:82, *v*/*v*), a detection wavelength of 355 nm, and an injection volume of 10 μL at room temperature. For the detection of TC, the operation conditions were a flow rate of 1.0 mL/min, a mobile phase of acetonitrile/water (15:85, *v*/*v*, pH of 2.95 adjusted by citric acid), a detection wavelength of 350 nm, and an injection volume of 10 μL at room temperature. The eluted times for the three chemicals were all almost 6 min.

### 5.4. MC-LR Analysis

MC-LR is a frequently encountered MC variant containing the l-amino acids leucine (L) and arginine (R). We previously found that MC-LR is released from cells after 48 h of exposure to glyphosate and diclofop acid [32,40]. Therefore, this time point has also been chosen in the present work. In our future work, MC-LR levels throughout the entire exposure period will be analyzed. Thirty milliliter of cells and culture media from cultures which were exposed to different concentrations of the three chemicals for 48 h (with the same initial conditions as growth inhibition test) were separated by filtration through a 47-mm GF/C filter (0.45 μm, Whatman, Maidstone, Kent, UK). Aqueous filtrates, containing the extracellular MC-LR were directly applied to the MC-LR ELISA kit (Express Technology Co., Ltd., Beijing, China), following the manufacturer’s protocols. The samples were diluted with PBS at least for 8-fold before detection, to make fit in the detection range (0–4 μg/L). The microtiter plates were read at 450 nm in a Microplate Reader (BioTek, Winooski, VT, USA), and the concentrations of the extracellular MC-LR equivalents were calculated. The detection limit is 0.05 μg/L. Since ELISA antibodies are not specific of MC-LR (MC-RR and MC-YR can also be detected) and, normally ELISA results are expressed as “MC-LR equivalents”.

The filters with the attached cyanobacterial cells were sliced with a sterile scalpel. The intracellular MC-LR from the filters was extracted with 15 mL of distilled water in glass tubes, immersed in ice and sonicated for 15 min (JY96-IIN Ultrasonic Cell Disruptor, Ningbo, China). After sonication, the mixture was stirred for 10 min at room temperature and centrifuged for 10 min at 12,000× *g*, and the supernatant was collected. The pellet was resuspended in 15 mL of distilled water and re-extracted [41]. The resulting solutions were then applied to the previously mentioned ELISA kit following the manufacturer’s protocols. The microtiter plates were read at 450 nm, and the concentrations of intracellular MC-LR equivalents were calculated. Triplicate analyses were performed, and the values were averaged. The values of intracellular/extracellular toxin per cell were calculated by the intracellular/extracellular toxin contents divided by cell density (living cells) at 48 h.

### 5.5. Data Analysis

Statistical analysis was performed using Origin 8.0 (Microcal Software, Northampton, MA, USA) and SPSS 16.0 (SPSS, USA) to determine the significance among the treatments. ANOVA was performed to determine the differences among different groups, and *p* < 0.05 was considered statistically significant. Multiple comparisons between the groups were performed using post hoc test with the LSD method.

## Figures and Tables

**Table 1:** EC_10_ and EC_20_ values at 96 h for CTC, OTC, and TC (mg/L).

EC_x_	CTC (mg/L)	OTC (mg/L)	TC (mg/L)
*r^2^*	0.92	0.98	0.88
EC_10_	1.86	3.02	0.63
EC_20_	4.09	4.86	1.58

**Table 2:** Growth parameters of *M. aeruginosa* exposed to different concentrations of CTC, OTC and TC using logistic regression.

Concentration (mg/L)	CTC			OTC			TC		
	*N*_F_^a^	*t*_c_	k ^b^	*N*_F_	*t*_c_	k	*N*_F_	*t*_c_	k
0	93.35	3.43	0.62 (A )	92.52	3.36	0.56 (A′)	92.52	3.36	0.56 (A″)
1	92.15	3.41	0.61 (A)	88.12	3.15	0.60 (B′)	72.34	2.85	0.51 (A″B″)
2	87.06	3.60	0.54 (AB)	81.82	3.11	0.60 (B′)	60.41	2.73	0.47 (A″B″)
5	90.48	3.65	0.56 (AB)	68.60	3.08	0.48 (C′)	55.95	2.52	0.45 (B″)
10	80.53	4.05	0.48 (B)	46.17	1.93	0.48 (C′)	38.53	1.15	0.43 (B″)
20	22.70	0.22	0.54 (AB)	-	-	-	-	-	-

^a^ Cell number counted with 10^5^/mL; ^b^ Parameter k is the mean of three replicates. Different capitalized letters behind the k-values indicate significant differences (*p* < 0.05) among different concentrations (A, B, C for CTC, A′, B′, C′ for OTC, and A″, B″, C″ for TC).
